# Management strategies for healthcare worker burnout in Sub-Saharan Africa: an integrative review

**DOI:** 10.3389/fpubh.2026.1779807

**Published:** 2026-03-24

**Authors:** Fikile Khangamwa-Singano, Victor Hamilton Singano, Prisca Kaunda, Redson Biswick Machongo, Yamikani B. Nkhoma-Mussa

**Affiliations:** 1 School of Nursing, University of Wisconsin-Milwaukee, Milwaukee, WI, United States; 2 Department of Midwifery, Kamuzu University of Health Sciences, Lilongwe, Malawi; 3 University of Global Health Equity, Kigali, Rwanda; 4 Faculty of Life and Health Sciences, Ulster University, Derry/Londonderry, United Kingdom; 5 School of Nursing, University of Wisconsin-Madison, Madison, WI, United States

**Keywords:** adaptive strategies, burnout, burnout management, maladaptive strategies, Sub-Saharan Africa

## Abstract

Burnout among healthcare workers is widespread across Sub-Saharan Africa. There is limited evidence on addressing burnout in these settings. Therefore, this integrative review aimed to identify, map, and synthesize the strategies to mitigate burnout among healthcare workers. We conducted a systematic search on PubMed, EBSCOhost, Web of Science, and Google Scholar on studies published from 2015 to 2025. We included studies if they examined burnout management strategies among healthcare workers in Sub-Saharan Africa. Studies that focused solely on burnout prevalence or determinants, without describing any management strategy, were excluded. We identified 10,057 articles, of which 3 were included. Additionally, 6 studies were identified through backward reference searching of included articles and supplementary Google Scholar. The studies were conducted in Uganda, Mozambique, Botswana, and South Africa. Sample sizes ranged from 10 to 1856 participants. Two overarching themes emerged: adaptive and maladaptive coping strategies. Evidence indicated that adaptive coping strategies, such as supportive supervision, workplace wellness activities, peer support, and culturally embedded coping practices, enhanced motivation, job satisfaction and emotional resilience. Although some strategies traditionally labeled as maladaptive, particularly self-distraction, may be associated with lower burnout levels in the short term, prolonged use may mask underlying stressors, reduce help-seeking behaviors, and increase vulnerability to chronic burnout. This review reveals that healthcare workers consistently employ a mix of adaptive and maladaptive coping strategies. Adaptive strategies were associated with reduced emotional exhaustion and improved resilience. Health systems should implement interventions that strengthen adaptive coping strategies to reduce emotional exhaustion and build resilience while addressing organizational conditions that perpetuate maladaptive coping.

## Introduction

Burnout is a major occupational hazard for healthcare workers worldwide, driven by persistent workplace stress that is not effectively managed (1, 2). As an occupational phenomenon, burnout is characterized by emotional exhaustion, depersonalization, and reduced personal accomplishment, which negatively affect mental health, job satisfaction, and quality of patient care (3, 4). Global evidence shows an increasing prevalence of burnout among healthcare workers. In the United States, burnout rose from 32% in 2018 to 46% in 2022, with nurses reporting some of the highest levels (5, 6). High workloads, emotional labor, and organizational pressures continue to fuel these trends (2).

Burnout is even more pronounced in Sub-Saharan Africa (SSA). Chronic staff shortages, high patient volumes, resource constraints, and limited institutional support create sustained pressure that heightens vulnerability to burnout (7). Estimates in SSA range from 34% (8) to 87% (9), reflecting chronic workforce shortages, high patient-to-provider ratios, limited resources, and weak support systems. In Tanzania and Malawi, high levels of burnout were reported 54.5 and 68%, respectively which are consistent with prevalence ranges observed across the Sub-Saharan African region (10, 11). Despite the high burden, most research in SSA focuses on identifying prevalence and associated factors rather than examining how burnout is being managed. There is a striking lack of intervention studies addressing burnout within the SSA region (9, 12).

Evidence remains scattered across small-scale studies and often lacks evaluation of effectiveness, highlighting the need for a comprehensive synthesis of available burnout management strategies in SSA (12). An integrative review is well-suited for this purpose because it accommodates diverse methodologies and allows for a holistic understanding of how healthcare workers manage or mitigate burnout across complex health system environments. Mapping these strategies supports the development of context-specific interventions that can strengthen resilience and improve provider wellbeing in resource-limited settings (13, 14).

## Objective

To identify strategies used to manage healthcare worker burnout in Sub-Saharan African countries.

## Research questions

1. What management strategies have been implemented to address healthcare worker burnout in Sub-Saharan Africa?
2. In which settings have burnout management strategies been implemented?
3. Which categories of healthcare workers have implemented or received burnout management strategies?
4. What are the outcomes associated with these strategies?

## Methods

### Review design

This integrative review was conducted in line with Whittemore and Knafl’s integrative review methodology guidelines for evidence synthesis, which supports the inclusion of diverse study designs (15). The review was reported according to the PRISMA Extension for Scoping Reviews (PRISMA-ScR) to ensure methodological transparency (16). Although this review followed Whittemore and Knafl’s integrative review methodology, the Population, Concept, and Context (PCC) framework was used pragmatically to define the review scope and eligibility criteria (Table 1). A protocol describing rationales and planned methods was registered under PROSPERO (17).

**Table 1:** Inclusion and exclusion criteria.

Domain	Inclusion criteria	Exclusion criteria
Population	Healthcare workers (nurses, midwives, doctors, clinical officers, allied professionals). Studies conducted in Sub-Saharan Africa.	Students, trainees, and non-clinical staff. Studies conducted outside Sub-Saharan Africa.
Phenomenon of interest	Studies examining burnout management strategies. Strategies including individual, organizational, community and cultural	Studies reporting only burnout prevalence or determinants without any management strategy.
Setting/context	Health facilities or health programs within Sub-Saharan Africa.	Non-healthcare settings.
Study design	Empirical quantitative, qualitative, mixed-methods, or implementation studies.	Editorials, commentaries, reviews, dissertations, conference, and abstracts without empirical data.
Publication characteristics	Published in English between 2015 and 2025.	Articles published before 2015 or not in English.
Outcome relevance	Reports interventions or strategies intended to manage or reduce burnout	Does not report any burnout management or coping approach

### Eligibility criteria

#### Information sources

Evidence was gathered from two phases of searching. The first phase used systematic searches in PubMed, EBSCOhost and Web of Science as these databases provide extensive indexing of biomedical, nursing, psychological, and multidisciplinary health research. The second phase used Google Scholar and reference list screening to identify additional eligible studies. This step was necessary because burnout-management research in Sub-Saharan Africa remains limited and fragmented (18, 19).

#### Search strategy

The search strategy was developed using four concept blocks combined with Boolean operators:

Healthcare worker terms: (“healthcare worker*” OR “nurse*” OR “doctor*” OR “midwife*” OR “clinical officer*”).
Burnout terms: (“burnout” OR “occupational stress” OR “job stress” OR “emotional exhaustion”).
Intervention/management strategy terms: (“intervention*” OR “management strateg*” “coping strateg*” OR “support program*” OR “wellness program*”).
Geographic terms (“Sub-Saharan Africa” OR Africa South of the Sahara): (“Malawi” OR “Uganda” OR “Botswana” OR “South Africa” OR “Kenya” OR “Tanzania” OR “Nigeria” OR “Zambia” OR “Ethiopia” OR “Zimbabwe”).

A comprehensive final search combined search terms from blocks 1, 2, 3, and 4 using AND: (Healthcare Worker Terms) AND (Burnout Terms) AND (Intervention Terms) AND (Geographic Terms). The Boolean operator OR was used to combine synonyms and related terms within the same concept to broaden the search and capture all relevant studies. Searches were limited to English-language publications from 2015 to 2025. Citation tracking of included studies was also undertaken to identify additional relevant articles.

#### Management of search results

All search results were imported into Rayyan for de-duplication and screening. Rayyan’s automated detection removed duplicate records (*n* = 58) before screening. After duplicate removal, FKS and VS independently screened titles and abstracts within Rayyan. Study selection occurred in two stages. First, 9,999 titles and abstracts were screened, and 9,933 were excluded for not meeting eligibility criteria, most commonly due to a lack of burnout management content. Second, 66 full texts were sought; however, 45 were not retrievable as they involved the wrong population or were conducted in sites outside the intended review context. Twenty-one full texts were screened, and 18 were further excluded. Fifteen studies had teachers as their study population (20, 21), population outside the SSA region (22, 23), and three had the wrong outcomes (24–26). Three studies met the criteria through database searching, and six additional studies were identified through Google Scholar and citation tracking (13, 14, 18, 19, 27, 28). Although the inclusion criteria specified studies published between 2015 and 2025, one study from 2014 was included because it reported a unique arts-based cultural approach, including songwriting and other creative activities used in the management of stress, grief, and emotional pain.

Database searches alone yielded a limited number of eligible studies, reflecting the scarcity of published evidence on burnout management strategies in Sub-Saharan Africa. To enhance search sensitivity and minimize the risk of missing relevant studies, supplementary searching was conducted using Google Scholar and citation tracking. Citation tracking was conducted by manually reviewing the reference lists of all included studies (backward reference searching) to identify potentially relevant articles not captured by database indexing. Each cited title was screened individually, and potentially relevant records were retrieved for abstract review against the predefined inclusion criteria. A structured Google Scholar search complemented this approach. Predefined burnout and management-related terms, combined with Boolean operators (AND, OR), were applied, consistent with the primary search strategy. Titles and abstracts were screened, followed by full-text assessment against the inclusion criteria. The two supplementary search strategies resulted in the identification of six additional studies that met the inclusion criteria and were incorporated into the review. This approach is recommended for reviews addressing complex, context-specific topics, where relevant evidence may be dispersed across diverse journals and inconsistently indexed in major databases (29). Disagreements during the inclusion process were resolved through discussion among the review team.

**Figure 1:**
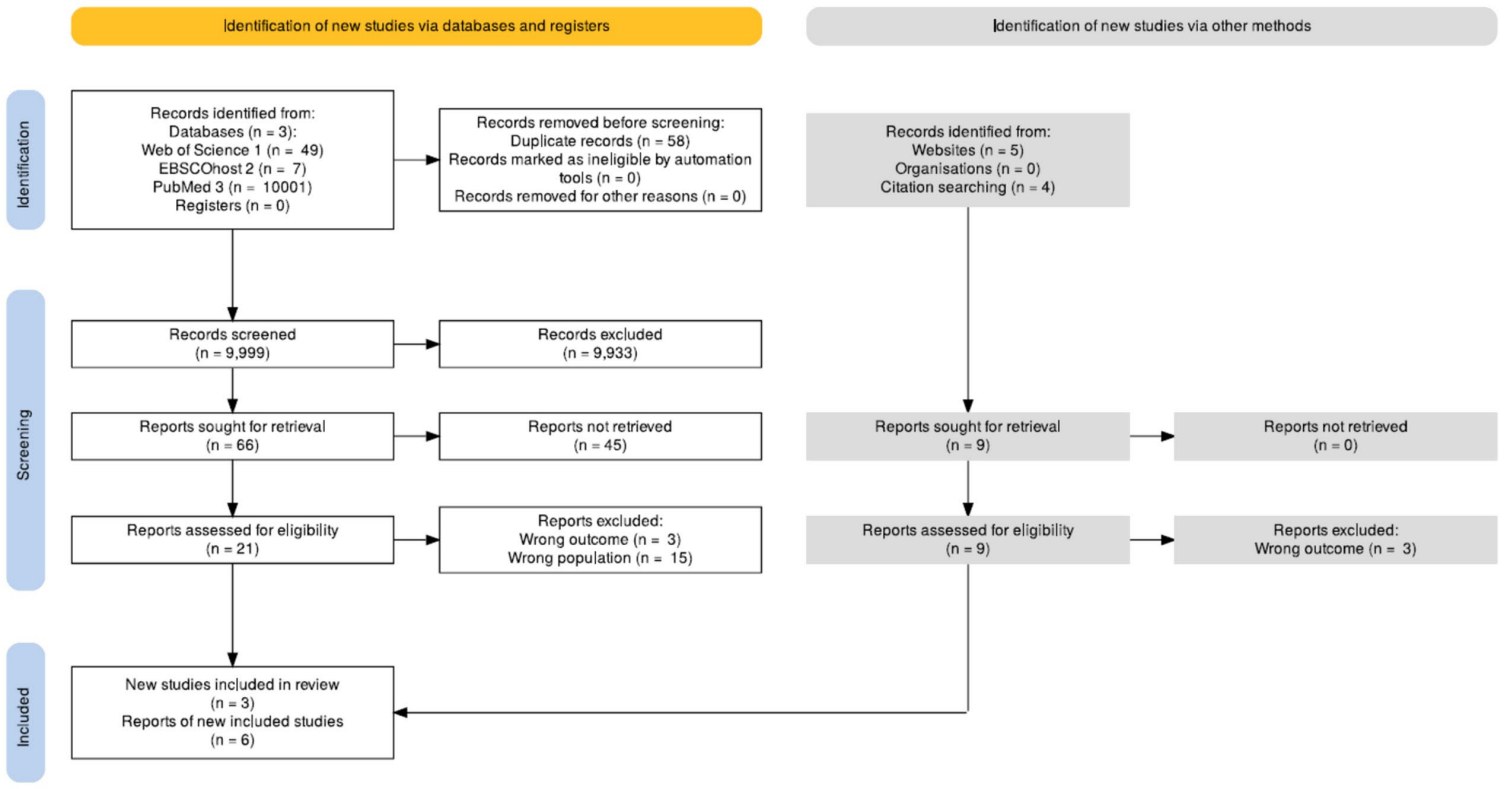
PRISMA diagram of included studies.

The final synthesis comprised nine studies. PRISMA-ScR flow diagram (Figure 1) summarized the selection process.

#### Data extraction, analysis, and synthesis

Data were extracted using a JBI-adapted extraction tool capturing study characteristics, setting, design, population, burnout-management strategy, measures, quality evaluation, and key findings (30). Quantitative data were summarized using frequencies, while qualitative data were analyzed thematically to identify recurring patterns across contexts. Results were then synthesized using a narrative and thematic integrative approach in accordance with Whittemore and Knafl’s integrative review methodology, involving data reduction, comparison, and synthesis across quantitative and qualitative studies. Findings were organized thematically to examine management strategies, contextual influences, and reported outcomes related to healthcare-worker burnout. Narrative synthesis was selected due to substantial heterogeneity in study designs, intervention types, outcome measures, and settings, which precluded statistical pooling of results.

#### Risk bias assessment

The cross-sectional studies by Moses et al. (28), Lee et al. (27), Ledikwe et al. (13), and Kabunga et al. (18),were appraised using the JBI Critical Appraisal Checklist for Analytical Cross-Sectional Studies (31). Overall, studies demonstrated moderate to high methodological quality, with the main source of bias relating to selection bias and residual confounding. Statistical analyses were generally appropriate for cross-sectional designs and outcomes were transparently reported (Table 2).

**Table 2:** Summary of risk of bias assessment of the included studies.

Author	Design	Appraisal tool	Selection and measurement bias	Confounding/causality	Statistical analysis and missing data	Reporting/other bias	Overall judgment
Moses et al. (28).	Cross-sectional	JBI, Analytical Cross-Sectional	Moderate, convenience sampling; low response rate Low, validated self-report tool	Moderate, partial adjustment; residual confounding likely	Low, utilized multiple regression. 1.9% missing data, which could unlikely affect findings	Low, methods and results were clearly reported	Moderate methodological quality: This study used validated measurement tools and appropriate statistical analyses. However, it was limited by selection bias from convenience sampling, a low response rate, reliance on self-reported data and the likelihood of residual confounding inherent to its cross-sectional design
Ledikwe et al. (13)	Cross-sectional	JBI, Analytical Cross-Sectional	Low, multistage sampling; good response rate of 73% Low, validated self-reported tool	Moderate, adjusted analyses; causality limited by design	Low appropriate analysis of covariance and analysis of variance used; however, the inability to infer causality, reliance on self-reported exposure and outcomes.	Low, transparent reporting	High methodological quality: Strengths include national sampling of purposively selected facilities, validated outcome measures, appropriate statistical adjustment, and a high response rate. Main limitations are the inability to infer causality, reliance on self-reported exposure and outcomes.
Lee et al. (27)	Cross-sectional	JBI, Analytical Cross-Sectional	Moderate, volunteer sampling Low, validated self-reported tool	Moderate, limited adjustment; residual confounding limited by design	Low, utilized multivariable logistic regression analysis to examine associations. Of the 154 participants, 122 completed surveys and were included in the final analyses	Low, methods and statistical analyses were generally clearly described.	Moderate methodological quality: Strengths are the use of validated instruments, clear variable definitions, and appropriate statistical modeling. Main limitations arise from volunteer sampling, self-reported data, and residual confounding inherent to the cross-sectional design.
Kabunga et al. (18)	Cross-sectional	JBI, Analytical Cross-Sectional	Moderate, unclear response rate; facility-based Low, validated self-report tool	High, no multivariable adjustment; therefore, confounding was not controlled	High, data were analyzed using descriptive statistics and Pearson/Spearman correlation; no multivariable analysis was conducted.	Low, methods and statistical analyses were generally clearly described	Moderate methodological quality: Strengths include the use of standardized, reliable instruments, clear outcome definitions, and a reasonably large multi-facility sample. However, the study is limited by its reliance on self-reported data and the absence of multivariable adjustment, meaning that the observed associations between coping strategies and burnout may be influenced by residual and unmeasured confounding
Repar and Reid (35)	Qualitative participatory	JBI – Qualitative	Some concerns, single hospice site however, took over 3 months, extended qualitative engagement.	N/A	Low, rich narrative evidence, multiple qualitative data sources interviews, field notes, reflective narratives, and creative artifacts. Member checking was not clearly reported	Some concerns, facilitator-researcher role overlap However, there were researchers’ strong reflexivity through extensive researcher field notes and reflective narration acknowledging the researcher’s positionality	Moderate methodological quality: This study has strong relational engagement, rich narrative data, and credible, well-grounded interpretations that show how arts-based encounters foster affirmation, emotional release, connection, agency, and personal transformation among hospice caregivers.
Oosthuizen et al. (33)	Qualitative phenomenology	JBI, Qualitative	Some concerns, small single-site sample; however, study had clear inclusion criteria	N/A	Low, strong thematic grounding, with thick descriptions.	Low researcher consistently examined and bracketed experiences throughout the research	High methodological quality: The study has a strong methodological alignment, rigorous data collection and analysis, and rich presentation of participant voices. Bracketing provided logical flow. The main limitations are the small, single-site sample, limiting transferability
Masoloko et al. (19)	Qualitative	JBI, Qualitative	Some concerns, single-site. However, had clear inclusion criteria.	N/A	Low, very rich verbatim quotations, data saturation was reported. Systematic thematic analysis was clearly reported.	Some concerns, positionality limited	High methodological quality: The design, sampling approach, data collection, and thematic analysis are appropriate and well-described, with good use of verbatim quotes and clear attention to trustworthiness. The main limitations are the small, single-site sample and limited reflexivity, which constrain transferability and leave some uncertainty about how researcher perspectives may have shaped the findings.
Scheunemann et al. (34)	Qualitative	JBI, Qualitative	Low, Multiple sites improving variation and Experiences were captured across different facilities	N/A	Low, quotations and thick contextual description Multiple data collection methods. Researchers positionality is clearly stated	Low, dual-coder validation with consensus. Constant reflexivity clearly stated	High methodological quality: Used the JBL qualitative Appraisal Tool, high qualitative rigor and coherent integration, with well-developed themes on multilevel coping supported by systematic coding and participant accounts.
Madede et al. (14)	Cluster-controlled trial	Cochrane RoB-2 (cluster)	High risk, non-random as districts were purposively selected. Baseline imbalances across arms	Some concerns, as participants were recruited after cluster allocation, which might have introduced baseline differences	Low risk, validated outcomes. Despite being a self-reported tool Outcomes were reported as intended, hence low risk High risk bias due to missing outcome data, substantial attrition from baseline to endline.	High risk, high attrition rates and a small sample. Some concerns related to deviations from the intended intervention were noted due to variability in supervision delivery across facilities.	Moderate methodological quality: The cluster-controlled trial was assessed as having some concerns to high risk of bias. Nonetheless, it provides useful exploratory evidence, particularly through qualitative findings that highlight perceived benefits of supportive supervision. Quantitative effects on burnout, work engagement, and job satisfaction were inconclusive. Non-random allocation, substantial attrition, small sample sizes, and the absence of cluster-adjusted analyses limit confidence in the effect estimates. Consequently, the findings should be interpreted and applied with caution, particularly when considering causal inferences or broader generalizations.

**Figure 2:**
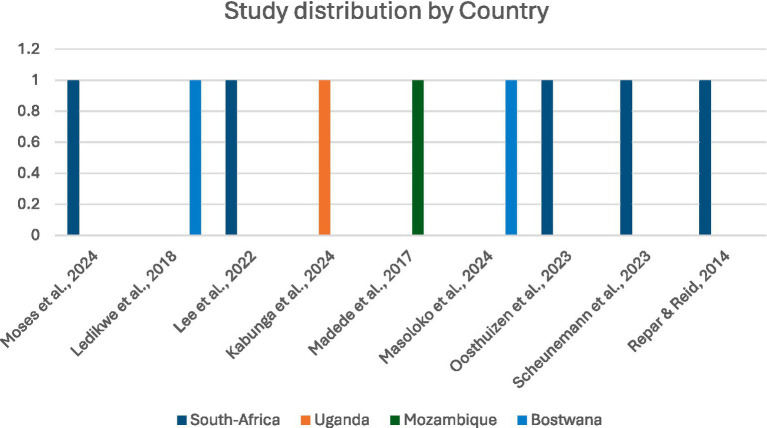
Distribution of studies across countries.

The cluster-controlled trial by Madede et al. (14) was assessed using the Cochrane Risk of Bias-2 tool for cluster trials and was judged to have a high overall risk of bias (32) and moderate methodological quality. Low quality stemmed from non-random allocation of clusters, substantial attrition between baseline and follow-up, small and unequal cluster sizes, and lack of statistical adjustment for clustering, all of which reduce confidence in the quantitative effect estimates. Nevertheless, the study provides valuable contextual and implementation-level insights into supportive supervision in real-world low-resource settings, complementing the quantitative evidence base (Table 2).

The qualitative studies, by Masoloko et al. (19), Oosthuizen et al. (33), Scheunemann et al. (34), and Repar et al. (35) were appraised using the JBI Critical Appraisal Checklist for Qualitative Research (36) and were generally of high credibility. These studies demonstrated strong alignment between aims, methodology, and thematic analysis, with findings grounded in rich participant quotations. However, they were limited by small, single-site samples and limited reflexive consideration of researcher influence, which primarily affects transferability rather than internal validity.

## Results

### Characteristics of included studies

**Figure 3:**
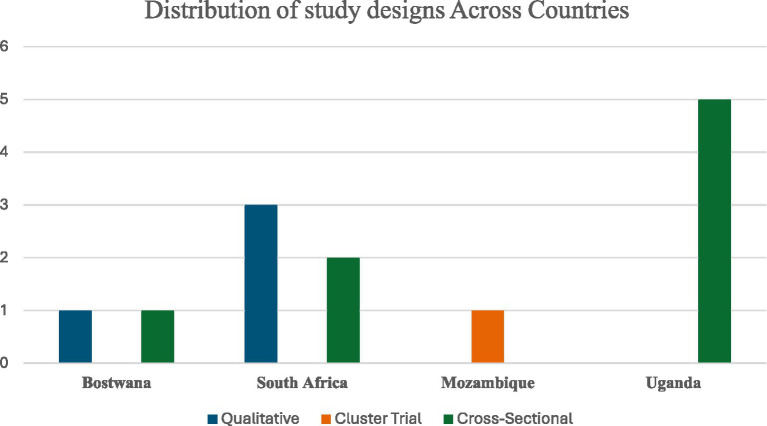
Distribution of study designs across countries.

**Figure 4:**
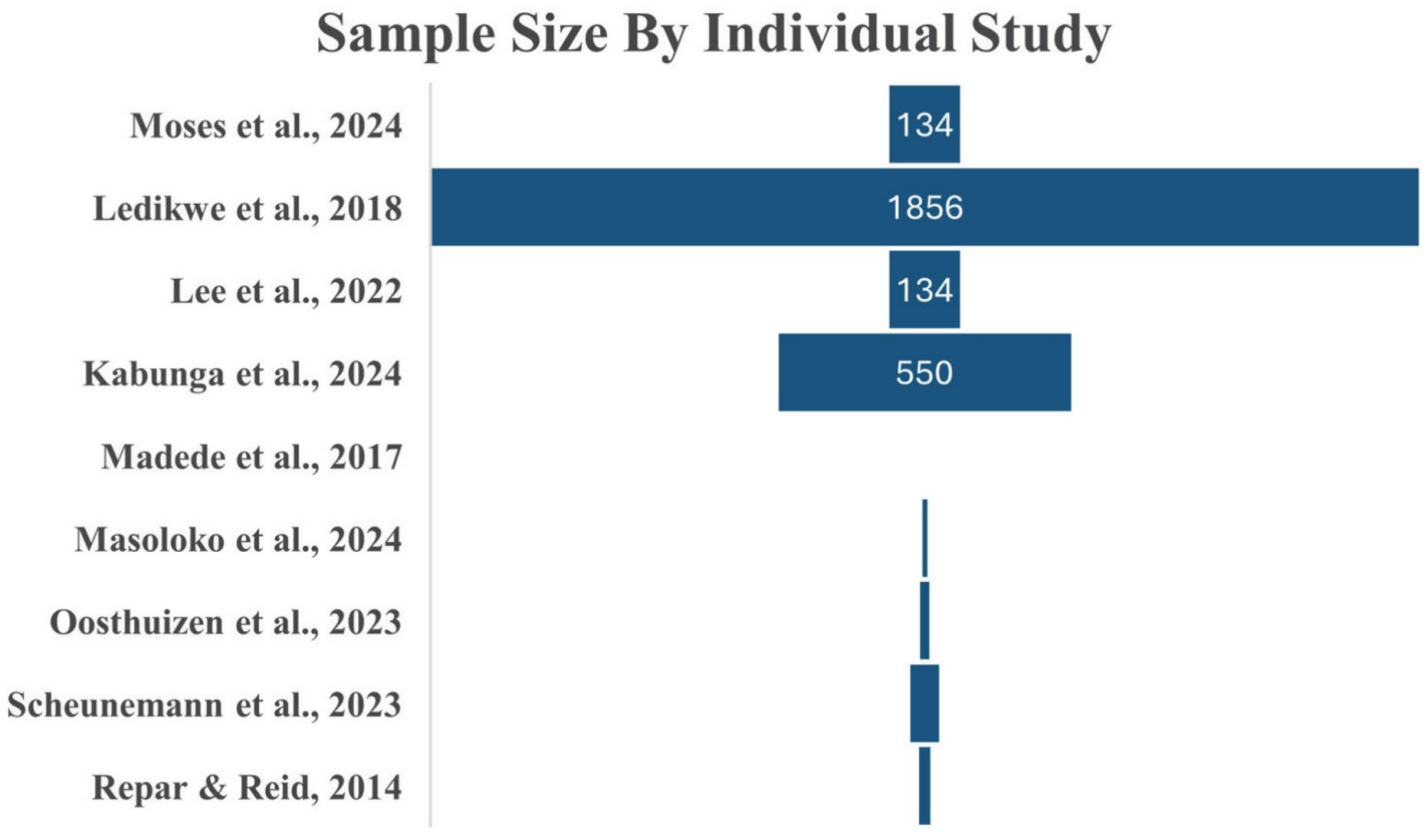
Sample size by individual studies.

The nine included studies were conducted in Botswana, Mozambique, South Africa, and Uganda, reflecting diverse clinical and psychosocial experiences across Sub-Saharan Africa (Figure 2). The studies employed a range of methodological approaches, including four qualitative studies, four cross-sectional studies, and one cluster-controlled trial (Figure 2). Sample sizes varied widely, from small qualitative groups of approximately 10 participants to large cross-sectional surveys of 1,856 healthcare workers (Figure 3). A total of 2,920 healthcare workers were included. Among studies that reported gender distribution, female healthcare workers represented a substantial proportion of respondents 1,631 (56%), although gender reporting was inconsistent across studies (Figure 4).

### Settings and healthcare worker cadres involved

Burnout management strategies have been implemented across a diverse range of healthcare settings in Sub-Saharan Africa (Table 3), involving multiple professional cadres. The studies were carried out across a wide range of healthcare settings, including public hospitals, community, rural, primary, secondary and tertiary health facilities, and national-level workforce initiatives. Cross-sectional studies (13, 14, 18, 28) primarily involved frontline clinical staff, including nurses, physicians, and allied health professionals such as social workers and occupational therapists, working in urban and rural hospitals. Qualitative studies (19, 33–35) captured the perspectives of community health workers, palliative care workers, and nurses working under chronic emotional and material constraints. Healthcare worker cadres ranged widely, from nurses, midwives, physicians, technicians, and community health workers to mixed multidisciplinary teams (Table 3). These studies demonstrate that burnout and its management strategies cut across multiple settings and professional groups, with nurses being the most represented.

**Table 3:** Characteristics of included studies.

Author	Cadres included	Type of healthcare facility/setting
Moses et al. (28)	Healthcare providers (mixed cadres doctors, nurses, allied workers, occupational and speech therapists)	Primary healthcare settings, clinics, community health centers and district hospitals
Ledikwe et al. (13)	Healthcare workers participating in workplace wellness programs (mixed cadres, doctors, nurses, administrators and other professions)	Public-sector facilities implementing wellness activities
Lee et al. (27)	Healthcare workers (nurses, support staff, other professions)	Public academic hospital
Kabunga et al. (18)	Healthcare workers (nurses, midwives, doctors, clinical officers)	Central Uganda health facilities
Repar and Reid (35)	Hospice caregivers (nurses and caregivers)	Hospice care settings
Masoloko et al. (19)	Nurses working in a psychiatric hospital	Psychiatric hospital
Oosthuizen et al. (33)	Female medical doctors	Public-sector medical facilities
Scheunemann et al. (34)	Psychiatric healthcare workers (nurses, clinicians, support staff)	Public psychiatric hospitals and specialized psychiatric hospitals, Gauteng Province
Madede et al. (14)	Health workers (nurses, doctors)	Primary healthcare facilities, Niassa Province

### Management strategies of healthcare worker burnout

This review intentionally differentiated between organizational/programmatic strategies and individual coping responses. Burnout is widely understood as arising primarily from chronic workplace stressors embedded within organizational systems rather than solely from individual vulnerability (3). Accordingly, organizational strategies target structural determinants such as workload, leadership practices, and supportive supervision, whereas individual coping responses reflect adaptive or maladaptive behaviors employed by healthcare workers to manage stress (2, 4). During data analysis, these individual responses were further synthesized and categorized by the review authors as adaptive or maladaptive burnout strategies to facilitate cross-study comparison and integration of findings as primary studies did not consistently apply this classification. Making this distinction explicit is important, as burnout is recognized as an occupational phenomenon driven largely by systemic and organizational factors (3), and excessive focus on individual coping may divert attention from these structural determinants (Table 4).

**Table 4:** Management strategies and key findings.

Author	Study design	Management strategies examined	Key findings			Remarks
			Outcomes	Adaptive strategies	Maldaptive strategies	
Moses et al. (28)	Cross-sectional	No direct intervention; examined organizational factors and coping influences on burnout.	High burnout was associated with workload, role conflict and resource shortages, weak organizational support and staffing gaps.	Multivariate analyses showed that better work demands, stronger management support, and clearer role definitions were each linked to meaningful improvements in Personal Accomplishment.	None	Highlights the need for organizational burnout management in rural areas.
Ledikwe et al. (13)	Cross-sectional	Workplace wellness programs (health promotion, exercise, counseling, staff activities).	High participation was associated with older age, being a doctor or other professional, working at hospitals or District Health Management Teams, working longer in health services or working longer at a facility. All associations remained significant in controlled analyses except cynicism. In unadjusted analyses,	Participation in wellness activities was associated with lower emotional exhaustion (*p* < 0.05) and higher job satisfaction. Overall job satisfaction assessed was significantly higher for health workers that participated in seven or more WWP activities, as compared with those who did not participate in any WWP activities (*p* < 0.001).	None	Demonstrates strong evidence for wellness programs in burnout reduction.
Lee et al. (27)	Cross-sectional	Mitigating factors: The study sought to identify factors and processes that contribute to the protection of HCWs from psychological distress.	The study found high levels of psychological distress among healthcare workers (57.4%) during the first wave of COVID-19 in South Africa, with distress strongly associated with perceived workplace risk related to COVID-19 exposure.	Healthcare workers who received relevant training and who reported supportive workplace relationships were significantly less likely to experience psychological distress.	None	Strengthening training and supportive organizational environments may enhance workforce resilience during future health emergencies.
Kabunga et al. (18)	Cross-sectional	Investigated burnout and coping strategies among healthcare professionals.	The study revealed a high prevalence of burnout, with 39.8% of participants experiencing significant levels.	The findings revealed that active coping, positive reframing and denial were negatively correlated with low burnout levels.	Maladaptive strategies: Dysfunctional coping, specifically self-distraction and denial, showed positive correlations with average and high burnout levels.	Coexistence of adaptive and maladaptive strategies underscores the need for targeted interventions that reinforce adaptive strategies.
Repar and Reid (35)	Qualitative	Arts-based therapeutic encounters (creative expression, singing, songwriting interventions).	Creative encounters fostered renewed hope, freedom, personal agency, and transformation for both caregivers and patients.	The study found that songwriting and other creative arts activities provided hospice caregivers with affirmation, emotional release, and acknowledgement of their experiences, helping them to process stress, grief and pain.	None	Unique evidence for arts-based stress reduction interventions, demonstrating that expressive arts can meaningfully enhance emotional wellbeing and clinical engagement.
Masoloko et al. (19)	Qualitative	Coping mechanisms: The study explored and described the coping mechanisms that nurses use to improve coping with burnout in a psychiatric hospital.	Four main themes emerged from the study, namely, coping with burnout in a psychiatric hospital, factors contributing to burnout among nurses, manifestations of burnout and suggestions to improve burnout.	Some of the coping sub-themes were debriefing with friends and colleagues, engaging in activities outside work like the gym, football, listening to music and lastly playing board games with patients. Participants made suggestions to improve burnout such as improving staff welfare issues, conducting staff meetings, recognition/support by management and increasing staff.	None	Although the study explored coping mechanisms, the underlying message was that nurses are largely left to manage burnout on their own through personal strategies.
Oosthuizen et al. (33)	Qualitative phenomenology	Coping strategies among female medical doctors.	Participants experienced burnout as emotional and physical exhaustion driven by heavy workloads and organizational constraints, including staff shortages, long hours, and limited institutional support.	Adaptive strategies such as peer support, spiritual support, time off, reflection, lifestyle adjustment, and positive reframing were used.	Maladaptive strategies: Some participants accepted the situation as beyond their control, though they remained feeling frustrated, which led to the adoption of a learned helplessness stance, self-neglect, withdrawal, denial and self-stimulation.	Reveals gender-specific burnout experiences and coping patterns.
Scheunemann et al. (34)	Qualitative	Coping during COVID-19: The study aimed to fill that gap by identifying and examining coping strategies and resources used by public healthcare workers in South Africa	We found that coping strategies spanned multi-level and multi-systemic efforts. Intrapersonal, interpersonal, material, and structural coping were mapped across individual, family, and hospital systems. , accommodation, and transport, although gaps in organizational support remained.	Psychiatric healthcare workers adopted multilevel coping strategies during the COVID-19 pandemic, combining intrapersonal approaches such as self-care, emotional regulation, spirituality, and meaning-making with interpersonal support from colleagues and family to manage stress. Coping was further shaped by organizational and environmental factors, including supportive supervision, workplace routines, and access to practical resources like protective equipment.	None	Demonstrates the value of multilevel coping strategies.
Madede et al. (14)	Cluster-controlled trial	The study aimed to evaluate an intervention designed to enhance healthcare workers’ motivation, engagement, job satisfaction, and retention.	Quantitative measures of job satisfaction, emotional exhaustion, and work engagement showed no statistically significant differences between baseline and end-line.	Adaptive strategies Qualitative findings indicated positive perceived effects of the intervention that were not captured by the survey measures. Health workers reported improvements in performance and motivation, attributed to supportive supervision. The intervention was also associated with increased participation and voice among health workers in intervention facilities.	None	Supportive supervision may produce meaningful improvements in health workers’ motivation, performance, and sense of voice.

Across the included studies, strategies addressing healthcare worker burnout were described using varied terms, including coping strategies, interventions, and management approaches. For analytic clarity, all were synthesized under the overarching category of management strategies and initially grouped as adaptive or maladaptive. Adaptive strategies referred to positive and constructive approaches aimed at preventing, reducing, or managing burnout. Maladaptive strategies described behaviors that may offer temporary psychological relief but are linked to worsening symptoms such as withdrawal, avoidance, denial, and self-distraction (18, 33, 37). Both structured organizational interventions and individual-level coping approaches were reported, many of which were associated with improved resilience, engagement, and professional functioning, consistent with occupational health literature on proactive burnout mitigation (2–4). Adaptive strategies were further organized into three levels: intrapersonal, organizational, and cultural or relational.

### Adaptive strategies

#### Intrapersonal

Across the reviewed studies, multiple adaptive strategies were identified as protective against burnout, enhancing psychological resilience among healthcare workers. Qualitative studies confirmed these patterns, showing that nurses and physicians who used humor, meaning-making, and self-reflection managed burnout more effectively, while those who suppressed emotions or detached themselves experienced greater emotional exhaustion (19, 33). In Uganda, active coping, where individuals take purposeful action to manage stress, was a key protective strategy. Kabunga et al. found that healthcare workers who used active coping reported significantly lower levels of burnout (18). Further, positive reframing, another adaptive strategy, was associated with reduced burnout in the same study, suggesting that cognitive reappraisal is beneficial in high-stress clinical environments.

#### Organizational

Planning and problem-focused coping were linked with lower burnout levels across several quantitative datasets (18, 28). In South Africa during the COVID-19 pandemic, team cohesion and supportive leadership emerged as particularly effective resilience mechanisms (27). Organizational strategies such as mentorship, wellness workshops, and structured supervision, as demonstrated in the Mozambique Support, Train and Empower Managers (STEM) intervention, enabled healthcare workers to build confidence and improve performance through constructive, on-site supervisory support (14). In Botswana and Mozambique, organizational structures, such as supportive supervision, clear communication, and participation in wellness activities, were associated with improved motivation, teamwork and job satisfaction (13, 14). Collectively, findings from Botswana and Mozambique indicate that supportive organizational leadership strengthens healthcare workers’ motivation, emotional wellbeing, teamwork, and job satisfaction. In addition, findings from South Africa, showed that staff felt less isolated and more emotionally supported when organizational leadership was present, responsive and communicative (33, 34). While these interventions improved morale, their measurable impact on burnout scores was sometimes limited, reflecting systemic constraints.

#### Cultural

Qualitative evidence underscored the role of culturally grounded expressive practices, such as singing, collective reflection, and participatory creative experiences, in processing emotions and maintaining a sense of connection and resilience. In the South African hospice setting, arts-based encounters strengthened social bonds, promoted emotional release, and cultivated a sense of affirmation and purpose, supporting sustained wellbeing (35). Culturally grounded coping, including spirituality, prayer, social connectedness and community identity, was widely used and experienced as protective, particularly where formal support systems were limited (19). Female clinicians drew on cultural expectations of resilience and collective identity to find meaning and sustain motivation (33).

#### Maladaptive strategies

In contrast, several studies revealed a high reliance on maladaptive strategies, particularly in settings marked by chronic resource shortages and overwhelming clinical demands. In central Uganda, burnout showed strong positive corelations with denial, and self-distraction, with denial demonstrating the strongest association with severe burnout (18). Healthcare workers experiencing high emotional exhaustion were significantly more likely to disengage cognitively, avoid confronting stressors, or emotionally distance themselves from their work.

Similarly, self-neglect, withdrawal, behavioral disengagement, and emotional suppression were commonly reported in both rural South Africa and Uganda (18, 33). South African rural clinicians frequently described avoidance behaviors and withdrawal as key responses to overwhelming stress, often reinforced by limited organizational support (18). In addition, a significant positive correlation was observed between high burnout levels and dysfunctional coping, particularly self-distraction in Uganda (18). In Botswana, emotional exhaustion was found to co-occur with low participation in wellness programs, suggesting that when support structures are limited or difficult to access, healthcare workers may resort to less helpful coping patterns (13). Studies during COVID-19 similarly demonstrated that weak infrastructure and insufficient institutional support intensified distress (27, 28, 34). These findings demonstrate that burnout interventions cannot succeed without parallel improvements in systemic capacity.

## Discussion

This integrative review synthesized nine studies exploring how healthcare workers in Sub-Saharan Africa manage or mitigate burnout. Collectively, the findings demonstrate that burnout management in the region is multifaceted, shaped by the interplay between organizational support, interpersonal relationships, individual burnout management strategies, and culturally grounded practices (12–14, 19). These findings echo broader regional evidence that burnout in Sub-Saharan Africa is driven primarily by structural conditions rather than individual weakness (12, 38). Chronic understaffing, heavy workloads, and limited resources restricted the feasibility or impact of burnout-management strategies (28, 34). Multiple systematic reviews and empirical studies show that organizational constraints undermine intervention effectiveness, even when strategies are well-designed (39, 40).

Organizational interventions emerged as essential components of burnout management. Supportive supervision programs improved teamwork, communication and provider motivation, even when changes in burnout scores were not statistically significant (14). Wellness programs in Botswana were associated with lower emotional exhaustion and higher job satisfaction (13). Global workplace intervention reviews also report that organizational strategies such as workload adjustment, job crafting and peer networks often lead to reductions in burnout and perceived stress and increases in resilience and engagement, reinforcing the importance of structural and team-level interventions to complement individual burnout management efforts (41). These findings are aligned with global evidence showing that organizational support reduces burnout risk and strengthens workforce resilience (2, 3).

Individual management strategies were also central to burnout management. Adaptive strategies were protective, while maladaptive approaches increased burnout (18). Qualitative studies reinforced these patterns and described coping as an ongoing negotiation shaped by personal beliefs, workload and emotional demands (19, 33). These findings demonstrated the multidimensional nature of burnout management strategies, which combined cognitive, behavioral and emotional strategies. Across multiple studies, maladaptive strategies such as denial, withdrawal, venting, disengagement, self-blame, and self-distraction were consistently associated with higher psychological distress, increased burnout, and poorer emotional and occupational outcomes among healthcare workers (18, 33). Similarly, findings from Romania and Ireland reinforced that maladaptive management strategies such as denial, venting, self-distraction, disengagement, and substance use were associated with poorer mental health outcomes (42, 43). These maladaptive coping patterns can impair concentration and increase the risk of clinical errors, ultimately compromising the quality and safety of patient care (37).

Arts-based programs were shown to facilitate emotional expression and stress relief among hospice caregivers, demonstrating the potential of creative, low-cost interventions in resource-constrained settings (29). Likewise, psychiatric staff reported that open communication, shared reflection, and mutual support strengthened their coping capacity during the COVID-19 pandemic (21). Together, these findings highlight the importance of embedding psychosocial support into routine clinical practice to enhance staff morale and reduce burnout.

This review also highlights that socio-cultural and faith-based coping strategies play a significant role in burnout management across several contexts. Spirituality strengthened resilience (19). Similarly, in Taiwan, a study of 458 healthcare workers found that Christian and Catholic faith was linked to higher resilience, suggesting that spirituality can act as a meaningful buffer against occupational stress (44). Consistent with these findings, a systematic review of spiritual wellbeing and burnout similarly concluded that higher spiritual wellbeing is generally linked to lower burnout, suggesting that faith-based and meaning-centered interventions may be especially relevant in contexts where spirituality is integral to daily life (45).

Finally, structural and system-level barriers strongly shaped burnout management. Studies from rural South Africa showed that burnout was highest in facilities with severe staff shortages, high workloads, role conflict, and limited organizational support (28). These findings parallel global evidence that burnout is deeply rooted in systemic conditions, including inadequate staffing, administrative pressures and resource shortages rather than individual deficits, underscoring the importance of organizational reform (3, 46). These regional patterns align with global evidence indicating that sustainable reductions in burnout depend on organizational and health system reforms, including adequate staffing, supportive leadership, and psychologically safe work environments, rather than relying solely on individual resilience or personal management strategies.

### Strengths and limitations

This integrative review was guided by Whittemore and Knafl’s integrative review framework and conducted in alignment with the Joanna Briggs Institute evidence synthesis methodology, which enabled the inclusion of diverse study designs and supported a comprehensive synthesis of quantitative and qualitative findings. The use of the PRISMA-ScR framework strengthened transparency and methodological rigor. Including studies from multiple Sub-Saharan African countries improved the relevance of findings across different health-system contexts. The review also captured a wide range of strategies, organizational support, emotional and psychological interventions, individual coping and culturally grounded practices, allowing a more holistic understanding of burnout management.

However, limitations were also noted. Although 10,057 records were initially identified, only three studies met the inclusion criteria, underscoring the nascent and limited state of research on burnout management in Sub-Saharan Africa. Evidence in the region remains scarce, and only a few intervention studies have been conducted, thereby limiting firm conclusions about effectiveness Many included studies were cross-sectional and qualitative studies, preventing causal inference and limiting generalizability beyond the specific study settings. A good number of full-text articles could not be retrieved; however, most were excluded due to an incorrect population, so the impact on the overall quality and completeness of the review is likely minimal.

### Implications for practice

The findings of this review highlight several important implications for health systems in Sub-Saharan Africa. Strengthening organizational support should be a priority, as interventions such as supportive supervision and workplace wellness activities have demonstrated positive effects on staff morale and resilience. In addition, culturally grounded approaches, such as spirituality and community support, should be incorporated into intervention design, as they align with local values and are more likely to be accepted and sustained. Importantly, very few intervention studies have been conducted in the region, despite high levels of burnout among healthcare workers. There is an urgent need for rigorous, context-specific intervention research that evaluates both short-term and long-term outcomes. Strengthening organizational readiness, improving staffing levels and addressing structural barriers will be essential to ensure the success and sustainability of any burnout-management initiative.

## Conclusion

This integrative review synthesized evidence on strategies used to manage burnout among healthcare workers in Sub-Saharan Africa. The findings showed that burnout management relied on organizational support, interpersonal relationships, individual burnout management strategies and culturally grounded practices. Although supportive supervision, wellness programs and creative or emotional support strategies demonstrated promise, the evidence base remained limited. Most interventions were small-scale, descriptive and lacked long-term evaluation. Despite this, the review highlighted clear opportunities to strengthen workforce resilience through context-appropriate, low-cost and system-supported interventions. More rigorous, sustained and multisectoral approaches are urgently needed to address burnout in a region experiencing some of the highest global burdens of workforce stress.
