# Correction: Management strategies for healthcare worker burnout in Sub-Saharan Africa: an integrative review

**DOI:** 10.3389/fpubh.2026.1844069

**Published:** 2026-04-16

**Authors:** Frontiers Production Office

**Affiliations:** Frontiers Media SA, Lausanne, Switzerland

**Keywords:** adaptive strategies, burnout, burnout management, maladaptive strategies, Sub-Saharan Africa

Affiliation 3, University of Global Health Equity, Kigali, Rwanda, was erroneously given as Kamuzu University of Health Sciences, Lilongwe, Malawi.

The city for affiliation 2 was erroneously given as Blantyre. The correct city is Lilongwe.

The original version of this article has been updated.

